# Machine Learning Patient-Specific Prediction of Heart Failure Hospitalization Using Cardiac MRI-Based Phenotype and Electronic Health Information

**DOI:** 10.3389/fcvm.2022.890904

**Published:** 2022-06-16

**Authors:** Aidan K. Cornhill, Steven Dykstra, Alessandro Satriano, Dina Labib, Yoko Mikami, Jacqueline Flewitt, Easter Prosio, Sandra Rivest, Rosa Sandonato, Andrew G. Howarth, Carmen Lydell, Cathy A. Eastwood, Hude Quan, Nowell Fine, Joon Lee, James A. White

**Affiliations:** ^1^Stephenson Cardiac Imaging Centre, University of Calgary, Calgary, AB, Canada; ^2^Division of Cardiology, Department of Cardiac Sciences, Libin Cardiovascular Institute of Alberta, Calgary, AB, Canada; ^3^Libin Cardiovascular Institute of Alberta, Calgary, AB, Canada; ^4^Department of Diagnostic Imaging, University of Calgary, Calgary, AB, Canada; ^5^Department of Community Health Sciences, Cumming School of Medicine, University of Calgary, Calgary, AB, Canada; ^6^Data Intelligence for Health Lab, Cumming School of Medicine, University of Calgary, Calgary, AB, Canada; ^7^Department of Cardiac Science, Cumming School of Medicine, University of Calgary, Calgary, AB, Canada

**Keywords:** cardiovascular magnetic resonance imaging, machine learning, heart failure hospitalization, prediction, systolic heart failure (HF)

## Abstract

**Background:**

Heart failure (HF) hospitalization is a dominant contributor of morbidity and healthcare expenditures in patients with systolic HF. Cardiovascular magnetic resonance (CMR) imaging is increasingly employed for the evaluation of HF given capacity to provide highly reproducible phenotypic markers of disease. The combined value of CMR phenotypic markers and patient health information to deliver predictions of future HF events has not been explored. We sought to develop and validate a novel risk model for the patient-specific prediction of time to HF hospitalization using routinely reported CMR variables, patient-reported health status, and electronic health information.

**Methods:**

Standardized data capture was performed for 1,775 consecutive patients with chronic systolic HF referred for CMR imaging. Patient demographics, symptoms, Health-related Quality of Life, pharmacy, and routinely reported CMR features were provided to both machine learning (ML) and competing risk Fine-Gray-based models (FGM) for the prediction of time to HF hospitalization.

**Results:**

The mean age was 59 years with a mean LVEF of 36 ± 11%. The population was evenly distributed between ischemic (52%) and idiopathic non-ischemic cardiomyopathy (48%). Over a median follow-up of 2.79 years (IQR: 1.59–4.04) 333 patients (19%) experienced HF related hospitalization. Both ML and competing risk FGM based models achieved robust performance for the prediction of time to HF hospitalization. Respective 90-day, 1 and 2-year AUC values were 0.87, 0.83, and 0.80 for the ML model, and 0.89, 0.84, and 0.80 for the competing risk FGM-based model in a holdout validation cohort. Patients classified as high-risk by the ML model experienced a 34-fold higher occurrence of HF hospitalization at 90 days vs. the low-risk group.

**Conclusion:**

In this study we demonstrated capacity for routinely reported CMR phenotypic markers and patient health information to be combined for the delivery of patient-specific predictions of time to HF hospitalization. This work supports an evolving migration toward multi-domain data collection for the delivery of personalized risk prediction at time of diagnostic imaging.

## Introduction

Heart failure (HF) is estimated to affect approximately 64 million people worldwide (1) and is associated with a high incidence of disease-related hospitalization (2). HF hospitalization is increasingly prioritized as an important clinical outcome by patients and healthcare organizations given strong associations with morbidity, mortality and dominant contribution to healthcare expenditures (3). In 2017 it was estimated that each HF hospitalization incurred a mean cost of $14,631 USD with 40% of patients readmitted within 90 days (4). Of all patient populations, those with systolic HF provide greatest contributions to HF hospitalization costs (2), justifying an expanding focus on this population for risk modeling. While the prediction of HF re-admission early following index HF hospitalization has been explored from administrative health data (5–7), risk models for incident HF hospitalization applicable to broader HF populations are required. The deployment of such models at time of diagnostic imaging, delivering descriptors of disease with opportunity for the capture of contextual health information, provides an attractive solution for personalized prediction modeling.

Cardiovascular magnetic resonance (CMR) imaging has become a routinely engaged test for the diagnosis and management of systolic heart failure. This has been justified by its versatility for the delivery of a broad range of phenotypic markers that accurately differentiate ischemic from non-ischemic etiologies (8–10), describe patterns of tissue injury (8), identify valvular pathology (11), and deliver reference standard quantification of chamber volumes, function, and ventricular mass (12, 13). While demonstrated to provide independent value for the prediction of composite outcomes (14–17), the combined value of CMR-reported phenotypic features and contextual patient health information to deliver personalized predictions of HF-related outcomes remains unexplored.

We hypothesized that CMR-reported markers of disease contextualized to patient-reported and EHR-derived markers of health can permit patient-specific predictions of time to HF hospitalization. To achieve this, we explored both machine learning (ML)-based modeling and competing risk Fine-Gray (FGM)-based risk modeling techniques for individualized predictions of time to HF hospitalization at time of CMR.

## Materials and Methods

### Dataset Available for Risk Modeling

CMR imaging data, patient-reported measures of health status and electronic health record (EHR) abstracted data was provided by the Cardiovascular Imaging Registry of Calgary (CIROC, NCT04367220). CIROC is a prospectively recruiting clinical outcomes Registry of the Libin Cardiovascular Institute engaging patients clinically referred for cardiac diagnostic imaging. Consenting patients undergoing CMR imaging between February 2015 and October 2019 for the evaluation of systolic HF and completing a minimum 1-year follow-up period were included. All data was collected at time of diagnostic test performance using a commercial workflow, data integration, and diagnostic test reporting software platform (cardioDI™, Cohesic Inc., Calgary).

Patients with chronic systolic HF resulting from ischemic cardiomyopathy or idiopathic non-ischemic cardiomyopathy were identified. All patients were required to have CMR-based confirmation of reduced global systolic function, defined as a left ventricular ejection fraction (LVEF) ≤ 50%. Recognizing the unique natural history of patients with specific non-ischemic cardiomyopathy states, all patients with confirmed cardiac amyloid, cardiac sarcoidosis, and hypertrophic cardiomyopathy were excluded. Patients with an acute cardiomyopathy state due to recent (within 90 days) acute coronary syndrome, takotsubo cardiomyopathy, per-partum cardiomyopathy, or viral infection (suspected or confirmed acute myocarditis) were also excluded. This established a final patient cohort with chronic systolic HF of either ischemic or idiopathic non-ischemic etiology. Ischemic cardiomyopathy (ICM) was defined by occurrence of prior myocardial infarction, percutaneous coronary intervention and/or coronary bypass surgery, or presence of ischemic (subendocardial) pattern injury on late gadolinium enhancement (LGE) imaging corresponding to one or more vascular territories. Patients not meeting this criterion were classified as idiopathic non-ischemic dilated cardiomyopathy. For patients who underwent multiple CMR studies, the index study was used for prediction modeling.

A total of 8,773 unique patients enrolled in the CIROC Registry were considered. Of these, 2,455 had an LVEF ≤ 50% by CMR. Following application of the inclusion and exclusion criteria, 1,775 unique patients satisfied cohort eligibility.

The study was approved by the University of Calgary Conjoint Health Research Ethics Board. All subjects provided written informed consent. All research activities were performed in accordance with the Declaration of Helsinki.

### Data Element Generation and Collection

#### Patient Reported Health Data

Patient health questionnaires were electronically deployed prior to each CMR examination to collect patient demographics, comorbid cardiac and non-cardiac illness, smoking, and alcohol history, patient-reported shortness of breath [based on New-York Heart Association (NYHA) classification], and HRQoL using the EQ-5D tool (18).

#### Cardiovascular Magnetic Resonance Imaging-Based Phenotype Data

CMR imaging was performed on 3 Tesla clinical scanners (Prisma or Skyra, Siemens Healthcare, Erlangen, Germany) using standardized imaging protocols inclusive of routine cine and LGE imaging techniques in sequential short-axis views and 2-, 3-, and 4-chamber long axis views. Quantitative image analysis was performed using standardized operating procedures developed according to guidelines of the Society for Cardiovascular Magnetic Resonance (19). Image analysis was performed using commercially available software (cvi**42**; Circle Cardiovascular Inc., Calgary) to obtain left ventricular (LV) and right ventricular (RV) volumes and function from semi-automated contour tracing of the endocardial and epicardial borders followed by manual adjustment. Papillary muscles were considered part of the LV mass. Maximal left atrial volume was assessed in the phase immediately prior to mitral valve opening using the bi-plane area-length method on matched 2- and 4-chamber cine images. All measurements were indexed to body surface area, where appropriate, using the Mosteller formula.

Standardized software was used to receive and code quantitative markers of chamber volumes and function, and to code disease-specific phenotypes (cardioDI™; Cohesic Inc., Calgary). LGE images were visually scored for the presence, extent, and pattern of fibrosis: the latter scored as subendocardial, mid-wall striae, right ventricular insertion site, mid-wall patchy, subepicardial, and diffuse patterns, as previously described (20, 21). Valvular pathology was coded based upon visually graded assessments of regurgitation and stenosis severity. The presence of pleural and pericardial effusions was routinely coded.

#### Electronic Health Record Abstracted Data

Electronic health information was abstracted from institutional EHR data warehouses and was inclusive of pharmacy, laboratory and ICD-10 coded diagnostic and procedural data. Historic data was abstracted at time of index CMR, and every 3-months perpetually thereafter. The primary clinical outcome of HF-related hospitalization was identified by ICD-10 coding registered in the Discharge Abstract Database System, using primary ICD-10 codes of I50.X. All documented events were manually adjudicated by medical chart review. Mortality data, used for competing risk analysis, was collected from Vital Statistics Alberta.

### Statistical Analysis

Continuous variables are reported as means ± standard deviation whereas categorical variables are expressed as counts with percentages. Comparison between groups for continuous variables were performed using a Student’s *t*-test or a Welch’s *t*-test where appropriate. Chi-squared tests without a Yates Correction and Fishers Exact tests were used for comparison between groups for categorical variables. Univariable CoxPH analysis of baseline variables was performed to identify associations with the primary outcome, this used for identifying candidate variables for FGM-based modeling.

### Risk Model Development

We aimed to develop and compare performance of machine learning (ML) and non-ML based modeling for the patient-specific prediction of time to HF hospitalization with reference comparison to a historic HF prediction model. As a ML-based approach we used Random Survival Forest (RSF) based modeling, this compared to a FGM-based model. For the development of our novel prediction models our study dataset was split into a 70% (*n* = 1,245) development and 30% (*n* = 530) holdout validation cohort, balanced for both event rate and follow-up duration. The development cohort was partitioned into five training and testing datasets for 5-fold cross-validation-based model development and selection. Within each cross-validation fold, missing data was independently imputed by multivariable feature imputation (Hmisc: aregImpute) (22). Manual variable reduction was executed to remove variables with a missingness rate > 15% and those believed to have poor generalizability to other clinical settings (i.e., unique to the local institution). This led to 63 consistently available disease phenotype (imaging) and patient health variables for the development of our risk models (Supplementary Table 1).

### Machine Learning Model Development and Performance Evaluation

For each development fold, 100 bootstrap samples with replacement were generated and 100 RSF models were trained for variable selection. These models were applied to out-of-bag data where variable importance was then assessed using permutation importance rank. The top 15 performing variables for each training fold were selected by their mean variable rank across all 100 out-of-bag datasets. A comprehensive grid search technique was used for hyperparameter tuning, as summarized in Supplementary Table 2. Optimized models in each training fold were applied to the test sets for final model evaluation and selection using time-dependent area under the curve (tAUC) and C-index. Models containing a range of 13–17 features were assessed in the test set, comparing tAUC and C-index to identify the optimal number of model features. The final model was then applied to the holdout dataset where performance was assessed using C-index, tAUC, average positive predictive value (PPV), average recall and F1 score. A model threshold for discriminating “High” from “Low” risk cohorts was then defined by observing the inflection point of observed events across deciles of predicted risk in the development cohort. Cumulative incidence plots accounting for competing events and stratifying patients by predicted risk category were generated. Calibration plots were generated by plotting mean difference in predicted and observed event rates for each decile of risk across 100 bootstrap replicates at 2 years. The Aalen-Johansen method was used to account for competing events.

### Competing Risk Fine-Gray-Based Models Risk Model Development and Performance Evaluation

For development of the FGM based model variable candidacy was defined by a threshold *p*-value of 0.1 in univariable analysis. Backward variable selection was performed to select features, based on order of variable exclusion. Highly correlated features were excluded using a threshold of a Pearson’s coefficient greater than 0.7. The competing risk FGM model was applied to generate coefficients for each variable that could then be used to estimate 90 day, 1 and 2-year probability of HF hospitalization for individual patients, as previously described (23). The test dataset was used to assess model performance for each development and test fold, resulting in five candidate risk models. C-index and tAUC were then used to select an optimal model for validation in the holdout dataset. Model performance in the holdout dataset was assessed using C-index, tAUC, average PPV, average recall and F1 score. Calibration was assessed using the method described above.

### MAGGIC Score-Based Risk Model Performance Evaluation

Originally developed for mortality prediction in systolic HF populations (24), the MAGGIC risk score served as the best available surrogate model for the estimation of HF outcomes in our referral population. MAGGIC risk scores were applied to the holdout cohort to provide matched assessments of performance vs. novel risk models.

All statistical analysis and modeling was performed in R version 4.0.3 and Python version 3.8.8 (25).

## Results

### Population Characteristics

Our chronic systolic HF population consisted of 1,775 unique patients with a mean age of 59 ± 13 years and 24% being female. Baseline clinical and CMR characteristics are summarized in Table 1. The population was composed of 52% ischemic cardiomyopathy and 48% non-ischemic dilated cardiomyopathy patients.

**TABLE 1:** Baseline clinical and CMR characteristics of the study cohort.

Data domain	Full population	Event –	Event +	*p*-value

	***n* = 1,775**	***n* = 1,442**	***n* = 333**	
**Patient reported health questionnaire**				
Age (years)	59 ± 13	58 ± 13	63 ± 13	**<0.0001**
Female, *n* (%)	418 (24)	336 (23)	82 (25)	0.6079
Obesity, *n* (%)	638 (36)	510 (35)	128 (38)	0.2925
NYHA class III or IV, *n* (%)	439 (25)	313 (22)	126 (38)	**<0.0001**
Atrial fibrillation, *n* (%)	322 (18)	247 (17)	75 (23)	**0.0213**
CAD, *n* (%)	397 (22)	305 (21)	92 (28)	**0.0106**
Diabetes, *n* (%)	346 (19)	251 (17)	95 (29)	**<0.0001**
Hypertension, *n* (%)	688 (39)	524 (36)	164 (49)	**<0.0001**
Hyperlipidemia, *n* (%)	382 (22)	290 (20)	92 (28)	**0.0026**
Peripheral arterial disease, *n* (%)	22 (1)	16 (1)	6 (2)	0.3034
Pulmonary hypertension, *n* (%)	26 (1)	14 (1)	12 (4)	**0.0003**
COPD, *n* (%)	87 (5)	56 (4)	31 (9)	**<0.0001**
Smoking, *n* (%)	340 (19)	279 (19)	61 (18)	0.6669
Mobility issues (EQ5D), *n* (%)	518 (29)	366 (25)	152 (46)	**<0.0001**
Anxiety/depression (EQ5D), *n* (%)	539 (30)	435 (30)	104 (31)	0.7033
Pain issues (EQ5D), *n* (%)	598 (34)	457 (32)	141 (42)	**0.0002**
Self-care issues (EQ5D), *n* (%)	172 (10)	121 (8)	51 (15)	**0.0001**
Issues with usual activity (EQ5D), *n* (%)	649 (37)	474 (33)	175 (53)	**<0.0001**
**Clinical patient history—administrative health data**				
Prior hospitalization—1 year, *n* (%)	894 (50)	669 (46)	225 (68)	**<0.0001**
Prior hospitalization—3 years, *n* (%)	1,169 (66)	895 (62)	274 (82)	**<0.0001**
Two weeks hospitalized in prior year, *n* (%)	248 (14)	160 (11)	88 (26)	**<0.0001**
Ischemic cardiomyopathy, *n* (%)	919 (52)	710 (49)	209 (63)	**<0.0001**
History of atrial fibrillation, *n* (%)	396 (22)	292 (20)	104 (31)	**<0.0001**
**CMR parameters**				
LVEF (%)	36 ± 11	37 ± 10	31 ± 11	**<0.0001**
LVESV index (mL/m^2^)	75 ± 36	71 ± 33	91 ± 42	**<0.0001**
LVEDV index (mL/m^2^)	113 ± 37	110 ± 35	127 ± 44	**<0.0001**
LVMass index (g/m^2^)	70 ± 21	68 ± 21	76 ± 23	**<0.0001**
RVEF (%)	47 ± 12	48 ± 11	44 ± 13	**<0.0001**
RVESV index (mL/m^2^)	45 ± 21	44 ± 20	51 ± 27	**<0.0001**
RVEDV index (mL/m^2^)	84 ± 24	83 ± 22	87 ± 30	**0.0260**
LA volume index (mL/m^2^)	44 ± 18	42 ± 17	50 ± 20	**<0.0001**
Presence of any LGE pattern, *n* (%)	1,064 (60)	831 (58)	233 (70)	**<0.0001**
Subendocardial pattern, *n* (%)	695 (39)	533 (37)	162 (49)	**0.0001**
Non-ischemic pattern, *n* (%)	679 (38)	542 (38)	137 (41)	0.2290
Midwall striae, *n* (%)	304 (17)	232 (16)	72 (22)	**0.0157**
RV insertion site, *n* (%)	392 (22)	302 (21)	90 (27)	**0.0159**
Midwall patchy, *n* (%)	119 (7)	96 (7)	23 (7)	0.8697
Subepicardial, *n* (%)	111 (6)	97 (7)	14 (4)	**0.0866**
Diffuse, *n* (%)	26 (1)	20 (1)	6 (2)	0.5701
**Medications**				
ACE inhibitor or ARB, *n* (%)	1,498 (84)	1,182 (82)	316 (95)	**<0.0001**
Anti-arrhythmic, *n* (%)	98 (6)	68 (5)	30 (9)	**0.0020**
Anti-coagulant, *n* (%)	547 (31)	393 (27)	154 (46)	**<0.0001**
Anti-platelet (non-ASA), *n* (%)	275 (15)	221 (15)	54 (16)	0.6857
ASA, *n* (%)	803 (45)	624 (43)	179 (54)	**0.0005**
Beta-blocker, *n* (%)	1,492 (84)	1,181 (82)	311 (93)	**<0.0001**
Calcium channel blocker (dihydropyradine), *n* (%)	186 (10)	142 (10)	44 (13)	0.0707
Calcium channel blocker (non-dihydropyridines), *n* (%)	56 (3)	46 (3)	10 (3)	0.8603
Digoxin, *n* (%)	138 (8)	92 (6)	46 (14)	**<0.0001**
Loop diuretic, *n* (%)	520 (29)	317 (22)	203 (61)	**<0.0001**
Thiazide diuretic, *n* (%)	136 (8)	108 (7)	28 (8)	0.5699
K-sparing diuretic, *n* (%)	718 (40)	529 (37)	189 (57)	**<0.0001**
Entresto, *n* (%)	178 (10)	142 (10)	36 (11)	0.5978
Glucose lowering, *n* (%)	310 (17)	212 (15)	98 (29)	**<0.0001**
Glucose lowering (DPP-4 inhibitors), *n* (%)	35 (2)	22 (2)	13 (4)	**0.0049**
Glucose lowering (SGLT 2 inhibitors), *n* (%)	38 (2)	32 (2)	6.0 (2)	0.6353
Insulin, *n* (%)	121 (7)	82 (6)	39 (12)	**0.0001**
Nitrates, *n* (%)	400 (23)	267 (19)	133 (40)	**<0.0001**
Statins, *n* (%)	1,005 (57)	778 (54)	227 (68)	**<0.0001**
Smoking cessation agents, *n* (%)	35 (2)	24 (2)	11 (3)	0.0525

*Variables are described for the full population and those with and without occurrence of the primary clinical endpoint of heart failure related hospitalization.*

*Quantitative data is presented as means ± standard deviation, qualitative data is presented as counts and percentages. History of Atrial Fibrillation variable is derived from administrative EHR data, Atrial Fibrillation variable is patient reported.*

*ACE, angiotensin-converting enzyme; ARB, angiotensin II receptor blocker; BSA, body surface area; CAD, coronary artery disease; COPD, chronic obstructive pulmonary disease; EDV, end-diastolic volume; EF, ejection fraction; ESV, end-systolic volume; ICM, ischemic cardiomyopathy; LA, left atrial; LGE, late gadolinium enhancement; LV, left ventricular; NYHA, New York Heart Association; RV, right ventricular. Bold values indicates p < 0.05.*

During a median follow-up period of 2.79 years (IQR: 1.59–4.04) 333 patients (19%) experienced the primary outcome of HF hospitalization. Ninety-five patients (5%) died, 40 of these (2%) dying without prior HF hospitalization.

No significant differences were observed between development and validation cohorts (Supplementary Table 3). In the development cohort, 233 patients (19%) experienced the primary outcome over a median follow-up period of 2.8 (IQR: 1.59–4.04) years. In the validation cohort 100 patients (19%) experienced the primary outcome over a median follow-up period of 2.74 (1.58–4.04) years.

### Machine Learning Risk Model Performance

**FIGURE 1:**
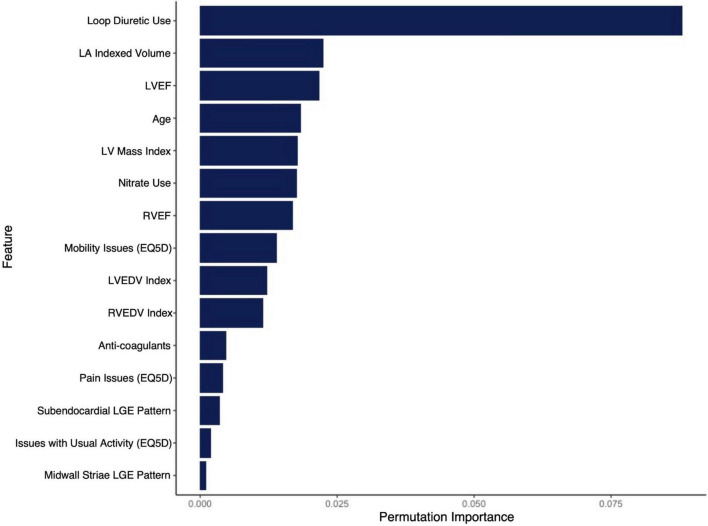
Mean permutation importance values over 100 bootstrap samples for the features included in the final CIROC-HF-RSF model.

**FIGURE 2:**
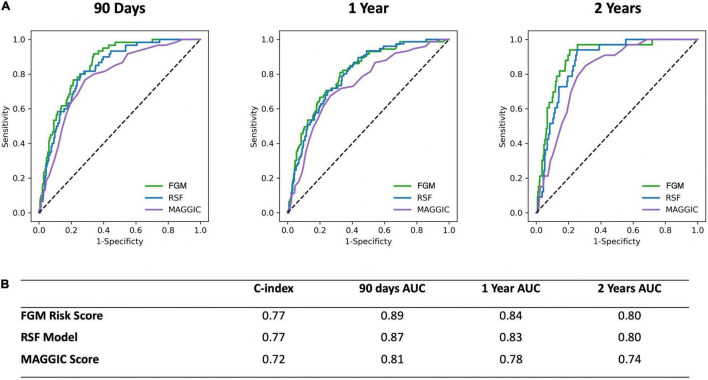
**(A)** Receiver operating characteristic curves for the CIROC-HF-RSF Model, CIROC-HF-FGM Risk Model, and modified MAGGIC risk score at 90 days, 1 and 2 years follow-up in the holdout cohort. **(B)** Summary of CIROC-HF-RSF model, CIROC-HF-FGM Risk Model, and modified MAGGIC risk score performance in the holdout cohort.

**FIGURE 3:**
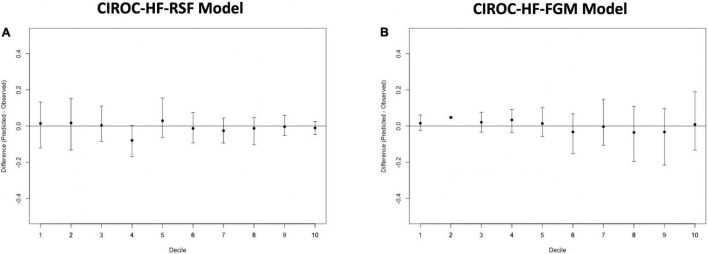
Calibration plots for **(A)** CIROC-HF-RSF risk model, and **(B)** CIROC-HF-FGM risk model for the prediction of HF hospitalization in the holdout cohort. Plots display difference between observed and expected event rates at each decile of risk. Confidence intervals are derived from 100 bootstrapped datasets.

**FIGURE 4:**
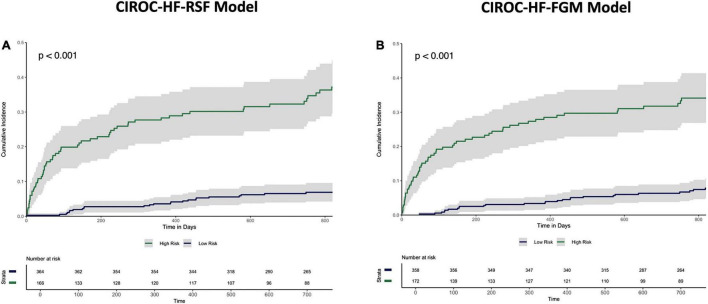
Cumulative incidence curves describing time to HF hospitalization in the holdout dataset stratified by “High-risk” vs. “Low-risk” classification by the **(A)** CIROC-HF-RSF model, and **(B)** CIROC-HF-FGM Risk Model.

The final RSF risk model contained15 predictive variables, 9 of which were sourced from the CMR-reported phenotype. The variable selection process produced a model containing LVESVi, LVEDVi, LVEF, RVESVi, RVEDVi, and RVEF. Given that end systolic volumes are implicit in a model containing end diastolic volumes and ejection fraction, two predictive late gadolinium enhancement patterns (subendocardial and mid-wall striae) were added to the model and performance in the test datasets compared. The model containing LGE features achieved higher C-index and tAUC values and this feature set was subsequently used to train the final RSF model. The mean permutation importance of each selected variables is shown in Figure 1. In the holdout cohort, the RSF model achieved a C-index of 0.77 and provided 90-day, 1 and 2-year AUC values of 0.87, 0.83, and 0.80, respectively (Figure 2). The RSF model delivered a mean PPV of 0.50 with an F1 score of 0.60 with good calibration across all deciles of risk (Figure 3). We defined patients with risk estimates above the seventh-risk decile to be “high-risk,” these patients experiencing 66% of all observed outcomes in the holdout cohort. Cumulative incidence curves (Figure 4) for patients predicted to be “high-risk” vs. “low-risk” showed significantly higher occurrence of HF hospitalization. The respective event rates for high vs. low-risk cohorts were 19 vs. 0.6% (*p* < 0.0001) at 90 days; 28 vs. 4% (*p* < 0.0001) at 1-year; and at 35 vs. 7% (*p* < 0.0001) at 2-years.

### Competing Risk Fine-Gray-Based Models Model Performance

Similar to machine learning-based modeling, variables from all data domains were shown to provide value toward an optimal FGM-based model with respective associations summarized in Supplementary Table 4. In holdout validation CIROC-HF-FGM delivered a mean C-index of 0.77 with 90-day, 1 and 2-year tAUC’s of 0.89, 0.84, 0.80, respectively. The mean PPV and F1 score was 0.49 and 0.59, respectively (Figure 2). Similar to the ML-based model, patients with a predicted risk above the seventh-risk decile were defined as “high-risk.” High-risk patients experienced 62% of all observed outcomes in the holdout cohort, with cumulative incidence curves shown in Figure 4. The respective event rates for high vs. low-risk groups were 18 vs. 1% (*p* < 0.0001) at 90-days; 28 vs. 3% (*p* < 0.0001) at 1-year; and 35 vs. 7% (*p* < 0.0001) at 2-years.

### Comparison of CIROC-HF Risk Models to the MAGGIC Risk Score

Both CIROC-HF risk models were compared to the MAGGIC Risk Score (24) in the validation cohort. The MAGGIC Risk Score delivered a mean C-index of 0.72 with a respective 90-day, 1-year, 2-year tAUC’s of 0.81, 0.78, 0.74. Comparisons of tAUC performance between the CIROC-HF risk models and MAGGIC Risk Score are shown in Figure 2, demonstrating superior performance for both novel CIROC-HF models.

## Discussion

In this study we demonstrated the capacity of routinely reported CMR disease markers to be contextualized by patient health information at the time of diagnostic testing for delivery of patient-specific estimations of time to HF hospitalization. Our modeling identified unique and independent value from each of the imaging phenotype, patient-reported health, and EHR data domains; their collective availability permitting improved prediction performance vs. the MAGGIC Risk Score. Using our ML-based model, patients classified to the high-risk category experienced a 34-fold higher occurrence of HF hospitalization at 90-days, 8-fold at 1-year, and 5-fold at 2-years. To our knowledge, this represents the first validated model for the prediction of HF hospitalization in HF patients undergoing CMR imaging.

HF hospitalization risk models have, to date, focussed on the prediction of re-admission following index hospitalizations for acute decompensation (5–7, 26–28). These models have consistently focussed on data sourced from in-patient electronic health records to identify those at higher likelihood of re-admission, typically at 90-days. All have struggled to achieve the performance of models trained to predict mortality (29, 30), suggesting elevated need to consider patient-specific disease phenotypes. The latter concept was explored in a study of 3,189 HF in-patients where multi-domain phenotypic data, gathered from routine echocardiography reporting, enabled prediction of all-cause early re-hospitalization with higher predictive accuracy than prior administrative data supported models, achieving an AUC of 0.76 at 90-days (31). While demonstrating value from multi-domain imaging phenotypes, this study was limited to high-risk inpatient populations, preventing generalizability to those patients routinely encountered by diagnostic imaging services.

Supported by a prior study showing incremental value from ML-based modeling for the prediction of HF re-admission using EHR sourced data (32), we postulated similar performance gains in our referral population. In contrast, we observed very similar performance for our modeled clinical outcome when compared to a FGM-based model provided matched multi-domain data resources. The exception was improved stability in time-dependent AUC seen using the ML-based approach at 2-years (Figure 2). However, a distinct advantage of ML-based modeling is its capacity to consider non-linear interactions between features without the limitations introduced by the proportional hazards assumption. Through this, we were afforded the opportunity to objectively evaluate the respective contributions of imaging phenotype, patient-reported health, and EHR-based markers have on the incident occurrence of HF hospitalization. As shown in Figure 1, we identified that current use of loop diuretics was the strongest contributor to model performance, followed by left atrial volume, LVEF, age and LV mass index. Other relevant features included volumetric markers of right ventricular health and patterns of myocardial fibrosis, these demonstrating the unique contributory value that CMR-based phenotyping can provide in this patient population. Collectively, these selected features appropriately represented phenotypic markers recognized to have strong predictive value in HF populations from prior studies (14, 15, 17, 20, 21, 33–46).

The capacity of contextual patient health information to contribute value for HF hospitalization prediction has been previously reported (47–49). To our knowledge, our current study is the first to describe the routine clinical deployment of patient-reported health questionnaires at time of diagnostic imaging for the delivery of this unique data domain. Of the top fifteen variables selected by our ML-based model, three were selected from the EQ-5D health related quality of life instrument (18). This demonstrates strong value from the synchronous capture of patient-reported measures of health at time of diagnostic testing, features recognized to be critical for the optimal prediction of HF-related events (29).

## Limitations

This study was executed at a single tertiary care healthcare institution and therefore requires external site validation prior to deployment beyond the local environment. This is of particular importance for unique clinical environments that may be exposed to a local referral bias in diagnostic testing or have altered socio-demographic profiles. While systematically explored, we did not report results of other classification-based ML techniques for event prediction at specific time-points given lower performance metrics. Due to lack of routine performed surrounding the time of CMR imaging, we were unable to consider BNP or NT-proBNP values into our predictive models. In addition, given the high engagement of private out-patient echocardiography laboratories in clinical practice, direct comparison to models trained from echocardiographic variables in the same patient population was not feasible. Advanced CMR based markers of myocardial disease of recognized value, such as tissue mapping (50), were not undertaken in this large cohort study given desire for generalizability to routine practice and a high degree of vendor and hardware dependence for such measures.

## Conclusion

In this study we developed and validated a machine learning based model for the prediction of time to HF hospitalization in systolic HF patients undergoing CMR. Our study was focussed on demonstrating the respective value provided by imaging phenotypes, patient-reported measures of health, and EHR-sourced data for the delivery of personalized HF predictions. Overall, our study supports strong value provided by the routine capture of multi-domain health data resources at time of diagnostic imaging, this approach facilitating the implementation of personalized outcome prediction.

## Data Availability Statement

The raw data supporting the conclusions of this article will be made available by the authors, upon appropriate request.

## Ethics Statement

The studies involving human participants were reviewed and approved by the University of Calgary Conjoint Health Research Ethics Board. The patients/participants provided their written informed consent to participate in this study.

## Author Contributions

AC performed all data analysis, modeling, and manuscript authorship. JW conceived, designed, edited, and finalized manuscript content. SD developed data structures and data matching for the CIROC Registry. DL, YM, JF, SR, RS, EP, and AS assisted in data collection and adjudication. NF, CL, AH, HQ, and CE performed study design and manuscript review. JL assisted in data modeling. All authors contributed to the article and approved the submitted version.

## Conflict of Interest

JW, AH, and JF were shareholders in Cohesic Inc. JW has received research funding from Siemens Healthineers, Circle Cardiovascular Inc., Pfizer Inc. AH received funding from Amgen. JW was Chief Medical Officer of Cohesic Inc. The remaining authors declare that the research was conducted in the absence of any commercial or financial relationships that could be construed as a potential conflict of interest.

## Publisher’s Note

All claims expressed in this article are solely those of the authors and do not necessarily represent those of their affiliated organizations, or those of the publisher, the editors and the reviewers. Any product that may be evaluated in this article, or claim that may be made by its manufacturer, is not guaranteed or endorsed by the publisher.

## Abbreviations

CIROC: Cardiovascular Imaging Registry of Calgary
CMR: Cardiovascular Magnetic Resonance Imaging
CoxPH: Cox Proportional Hazard
EHR: Electronic Health Record
FGM: Fine-Gray (Model)
HF: Heart Failure
HR-QOL: Health Related Quality of Life
LGE: Late Gadolinium Enhancement
LVEF: Left Ventricular Ejection Fraction
ML: Machine Learning
PPV: Positive Predictive Value
RSF: Random Survival Forest
tAUC: Time Dependent Area Under the Receiver Operating Characteristic Curve.
