# Supplementary material for: Machine Learning Patient-Specific Prediction of Heart Failure Hospitalization Using Cardiac MRI-Based Phenotype and Electronic Health Information
Source: Front Cardiovasc Med. 2022 Jun 16;9:890904. doi: 10.3389/fcvm.2022.890904 (PMC9245012; doi:10.3389/fcvm.2022.890904)
Supplement: Supplementary file 1 [file Data_Sheet_1.docx]

**SUPPLEMENTAL MATERIALS**

**Supplemental Table 1:** Sixty-three candidate variables provided for model development from the three data domains of imaging, patient health questionnaire and electronic health record.

| **Imaging**  **(n=16)** | **Patient Health Questionnaire (n=18)** | **Electronic Health Record**  **(n=29)** |
| --- | --- | --- |
| LVESV index | Patient Age | Ischemic Cardiomyopathy ^†^ |
| LVEDV index | Birth sex | Atrial Fibrillation by ECG, Holter or ICD-10 coding ^††^ |
| LVEF | Obesity (defined by BMI) | Any hospitalization in past year |
| RVESV index | NYHA Class III or IV | Any hospitalization in past 3 years |
| RVEDV index | Current Smoker | Any hospitalization ≥14 days duration in past year |
| RVEF | Atrial Fibrillation | Active medications |
| LA volume index | Coronary artery disease | ACE Inhibitor or ARB |
| LV Mass index | Diabetes | Oral Anti-coagulants |
| Presence of any LGE | Hypertension | Anti-arrhythmic |
| Subendocardial LGE Pattern | Hyperlipidemia | ASA |
| Mid-wall Striae LGE Pattern | Peripheral Artery Disease | Anti-platelet (non-ASA) |
| RV Insertion Site LGE Pattern | Pulmonary Hypertension | Beta Blocker |
| Mid-wall Patchy LGE Pattern | COPD | CCB (Dihydropyradine) |
| Subepicardial LGE Pattern | EQ5D variables: | CCB (Non-Dihydropyradine) |
| Diffuse LGE Pattern | Mobility Issues | Loop Diuretic |
| Presence of any non-ischemic LGE Pattern | Anxiety or Depression | K-Sparing Diuretic |
|  | Pain Issues | Thiazide Diuretic |
|  | Self-care Issues | Digoxin |
|  | Issues with Usual Activities | Entresto |
|  |  | Glucose Lowering (any) |
|  |  | Glucose Lowering  (Alphaglucosidase) |
|  |  | Glucose Lowering (SGLT2) |
|  |  | Glucose Lowering (DPP4) |
|  |  | Insulin |
|  |  | Ivabradine |
|  |  | Lipid Lowering (Statin) |
|  |  | Lipid Lowering (PCSK9) |
|  |  | Lipid Lowering (Other) |
|  |  | Nitrates |
|  |  | Smoking Cessation medication |
| **Abbreviations:** ACE, angiotensin-converting enzyme; ARB, angiotensin II receptor blocker; BSA, body surface area; CAD, coronary artery disease; CCB, Calcium Channel Blocker; COPD, chronic obstructive pulmonary disease; CMR, Cardiac Magnetic Resonance; DPP4, Dipeptyl-peptidase 4; EDV, end-diastolic volume; EF, ejection fraction; ESV, end-systolic volume; ICM, ischemic cardiomyopathy; LA, left atrial; LGE, late gadolinium enhancement. LV, left ventricular; NYHA, New York Heart Association; PCSK9, Proprotein convertase subtilisin/kexin type 9; RV, right ventricular, SGLT2, Sodium-glucose transport protein2. ^†^ Defined by combination of CMR referral indication, patient reported previous CAGB or PCI procedures and presence of subendocardial LGE pattern on the CMR images ^††^ I48.0-I48.2, I48.9. | | |

**Supplemental Table 2:** Nested hyperparameter grid search parameters.

| **Variable** | **Tested Range** | **Selected Value** |
| --- | --- | --- |
| Max Depth | 3, 5, 10, 20 | **5** |
| Max Features | sqrt(N) | **sqrt(N)** |
| Minimum Cases in Leaf | 5, 10, 15 | **5** |
| Minimum Samples for Split | 2, 5, 10 | **2** |
| Number of Trees | 10, 50, 100, 200, 400, 600, 800, 1000 | **50** |

**Supplemental Table 3**: Baseline clinical and CMR characteristics for the study cohort. Variables are described for the full population and those in the development and testing cohorts.

|  | **Full Population**  **n = 1775** | **Development**  **n = 1245** | **Validation**  **n = 530** | **p-value** |
| --- | --- | --- | --- | --- |
| **Patient Health Questionnaire** | | | | |
| Age (years) | 59 ± 13 | 59 ± 14 | 59 ± 13 | 0.7529 |
| Female, n (%) | 418 (24) | 294 (24) | 124 (23) | 0.9210 |
| Obesity, n (%) | 638 (36) | 438 (35) | 200 (38) | 0.3046 |
| NYHA Class III or IV, n (%) | 439 (25) | 316 (25) | 123 (23) | 0.3313 |
| Atrial Fibrillation, n (%) | 322 (18) | 217 (17) | 105 (20) | 0.2334 |
| CAD, n (%) | 397 (22) | 281 (23) | 116 (22) | 0.7518 |
| Diabetes, n (%) | 346 (19) | 241 (19) | 105 (20) | 0.8252 |
| Hypertension, n (%) | 688 (39) | 472 (38) | 216 (41) | 0.2605 |
| Hyperlipidemia, n (%) | 382 (22) | 273 (22) | 109 (21) | 0.5229 |
| Peripheral arterial disease, n (%) | 22 (1) | 17 (1) | 5 (1) | 0.4620 |
| Pulmonary Hypertension, n (%) | 26 (1) | 21 (2) | 5 (1) | 0.2329 |
| COPD, n (%) | 87 (5) | 54 (4) | 33 (6) | 0.0916 |
| Smoking, n (%) | 340 (19) | 251 (20) | 89 (17) | 0.0989 |
| Mobility Issues (EQ5D), n (%) | 518 (29) | 377 (30) | 141 (27) | 0.1188 |
| Anxiety/Depression (EQ5D), n (%) | 539 (30) | 387 (31) | 152 (29) | 0.3132 |
| Pain Issues (EQ5D), n (%) | 598 (34) | 415 (33) | 183 (35) | 0.6259 |
| Self Care Issues (EQ5D), n (%) | 172 (10) | 125 (10) | 47 (9) | 0.4449 |
| Issues with Usual Activity (EQ5D), n (%) | 649 (37) | 469 (38) | 180 (34) | 0.1376 |
| **Clinical History (Administrative Data)** | | | | |
| Prior Hospitalization – 1 Year, n (%) | 894 (50) | 637 (51) | 257 (48) | 0.3024 |
| Prior Hospitalization – 3 Years, n (%) | 1169 (66) | 827 (66) | 342 (65) | 0.4404 |
| Two Weeks Hospitalized in Prior Year, n (%) | 248 (14) | 182 (15) | 66 (12) | 0.2284 |
| Ischemic Cardiomyopathy, n (%) | 919 (52) | 655 (53) | 264 (50) | 0.2801 |
| History of Atrial Fibrillation, n (%) | 396 (22) | 279 (22) | 117 (22) | 0.8770 |
| **CMR Parameters** | | | | |
| LVEF (%) | 36 ± 11 | 36 ± 10 | 36 ± 1 | 0.6959 |
| LVESV index (mL/m²) | 75 ± 36 | 75 ± 36 | 75 ± 36 | 0.8542 |
| LVEDV index (mL/m²) | 113 ± 37 | 113 ± 37 | 113 ± 38 | 0.7367 |
| LV Mass index (g/m²) | 70 ± 21 | 70 ± 21 | 70 ± 21 | 0.7713 |
| RVEF (%) | 47 ± 23 | 47 ± 23 | 47 ± 12 | 0.7438 |
| RVESV index (mL/m²) | 45 ± 21 | 45 ± 12 | 46 ± 21 | 0.8174 |
| RVEDV index (mL/m²) | 84 ± 24 | 84 ± 24 | 84 ± 24 | 0.8646 |
| LA Volume index (mL/m²) | 44 ± 18 | 43 ± 17 | 44 ± 19 | 0.2868 |
| Presence of any LGE Pattern, n (%) | 1064 (60) | 753 (60) | 311 (59) | 0.4781 |
| Subendocardial Pattern, n (%) | 695 (39) | 499 (40) | 196 (37) | 0.2209 |
| Non-Ischemic Pattern, n (%) | 679 (38) | 473 (38) | 206 (39) | 0.7282 |
| Midwall Striae, n (%) | 304 (17) | 211 (17) | 93 (18) | 0.7590 |
| RV Insertion Site, n (%) | 392 (22) | 266 (21) | 126 (24) | 0.2630 |
| Midwall Patchy, n (%) | 119 (7) | 88 (7) | 31 (6) | 0.3472 |
| Subepicardial, n (%) | 111 (6) | 78 (6) | 33 (6) | 0.9755 |
| Diffuse, n (%) | 26 (1) | 16 (1) | 10 (2) | 0.3343 |
| **Medications** | | | | |
| ACE Inhibitor or ARB, n (%) | 1498 (84) | 1058 (85) | 440 (83) | 0.2975 |
| Anti-Arrhythmic, n (%) | 98 (6) | 67 (5) | 31 (6) | 0.6931 |
| Anti-Coagulant, n (%) | 547 (31) | 377 (30) | 170 (32) | 0.4537 |
| Anti-Platelet (non-ASA), n (%) | 275 (15) | 196 (16) | 79 (15) | 0.6555 |
| ASA, n (%) | 803 (45) | 572 (46) | 231 (44) | 0.3608 |
| Beta-Blocker, n (%) | 1492 (84) | 1049 (84) | 443 (84) | 0.7233 |
| Calcium Channel Blocker (Dihydropyradine), n (%) | 186 (10) | 140 (11) | 46 (9) | 0.1063 |
| Calcium Channel Blocker (non-Dihydropyridines), n (%) | 56 (3) | 36 (3) | 20 (4) | 0.3306 |
| Digoxin, n (%) | 138 (8) | 95 (8) | 43 (8) | 0.7282 |
| Loop Diuretic, n (%) | 520 (29) | 371 (30) | 149 (28) | 0.4751 |
| Thiazide Diuretic, n (%) | 136 (8) | 94 (8) | 42 (8) | 0.7861 |
| K-Sparing Diuretic, n (%) | 718 (40) | 514 (41) | 204 (38) | 0.2723 |
| Entresto, n (%) | 178 (10) | 133 (11) | 45 (8) | 0.1594 |
| Glucose Lowering, n (%) | 310 (17) | 217 (17) | 93 (18) | 0.9524 |
| Glucose Lowering (DPP-4 Inhibitors), n (%) | 35 (2) | 22 (2) | 13 (2) | 0.3416 |
| Glucose Lowering (SGLT 2 Inhibitors), n (%) | 38 (2) | 30 (2) | 8 (2) | 0.2305 |
| Insulin, n (%) | 121 (7) | 84 (7) | 37 (7) | 0.8578 |
| Nitrates, n (%) | 400 (23) | 282 (23) | 118 (22) | 0.8585 |
| Statins, n (%) | 1005 (57) | 694 (56) | 311 (59) | 0.2533 |
| Smoking Cessation Agents, n (%) | 35 (2) | 21 (2) | 14 (3) | 0.1855 |
| Quantitative data is presented as means ± standard deviation, qualitative data is presented as counts and percentages. **Abbreviations:** ACE, angiotensin-converting enzyme; ARB, angiotensin II receptor blocker; BSA, body surface area; CAD, coronary artery disease; COPD, chronic obstructive pulmonary disease; EDV, end-diastolic volume; EF, ejection fraction; ESV, end-systolic volume; ICM, ischemic cardiomyopathy; LA, left atrial; LGE, late gadolinium enhancement; LV, left ventricular; NYHA, New York Heart Association; RV, right ventricular. | | | | |

**Supplemental Table 4**: Univariable associations with heart failure hospitalization in the development cohort used for Development of the Cox Proportional Hazard risk model (CIROC-HF-CoxPH) (n = 1000)

|  |  |  |  |
| --- | --- | --- | --- |
| **Data Domain** | **Hazard Ratio** | **95% CI** | **p-value** |
| **Patient Health Questionnaire** |  |  |  |
| Age (years) | 1.02 | 1.01-1.03 | **0.0002** |
| Female, n (%) | 1.04 | 0.75-1.44 | 0.8200 |
| Obesity, n (%) | 1.15 | 0.86-1.56 | 0.3400 |
| NYHA Class III or IV, n (%) | 2.15 | 1.61-2.89 | **<0.0001** |
| Atrial Fibrillation, n (%) | 1.44 | 1.03-2.03 | **0.0340** |
| CAD, n (%) | 1.21 | 0.88-1.66 | 0.2400 |
| Diabetes, n (%) | 1.55 | 1.11-2.15 | **0.0090** |
| Hypertension, n (%) | 1.4 | 1.05-1.86 | **0.0200** |
| Hyperlipidemia, n (%) | 1.01 | 0.71-1.42 | 0.9600 |
| Peripheral arterial disease, n (%) | 2.5 | 0.93-6.74 | 0.0700 |
| Pulmonary Hypertension, n (%) | 2.56 | 1.20-5.45 | **0.0150** |
| COPD, n (%) | 2.71 | 1.67-4.40 | **0.0001** |
| Smoking, n (%) | 1.04 | 0.73-1.49 | 0.8300 |
| Problems with Mobility, n (%) | 2.28 | 1.72-3.03 | **<0.0001** |
| Anxiety/Depression, n (%) | 0.993 | 0.72-1.36 | 0.9600 |
| Problems with Pain, n (%) | 1.52 | 1.14-2.03 | **0.0042** |
| Problems with Self Care, n (%) | 1.81 | 1.22-2.69 | **0.0032** |
| Problems with Usual Activity, n (%) | 1.78 | 1.34-2.37 | **0.0001** |
| **Clinical History (Administrative Data)** | | | |
| Prior Hospitalization – 1 Year, n (%) | 2.62 | 1.91-3.57 | **<0.0001** |
| Prior Hospitalization – 3 Years, n (%) | 2.49 | 1.72-3.59 | **<0.0001** |
| Two Weeks Hospitalized in Prior Year, n (%) | 2.33 | 1.69-3.23 | **<0.0001** |
| Ischemic Cardiomyopathy, n (%) | 1.87 | 1.39-2.53 | **<0.0001** |
| History of Atrial Fibrillation, n (%) | 1.56 | 1.14-2.14 | **0.0050** |
| **CMR Parameters** | | | |
| LVEF (%) | 0.949 | 0.94-0.96 | **<0.0001** |
| LVESV index (mL/m²) | 1.01 | 1.01-1.01 | **<0.0001** |
| LVEDV index (mL/m²) | 1.01 | 1.00-1.01 | **<0.0001** |
| LV Mass index (g/m²) | 1.01 | 1.01-1.02 | **0.0001** |
| RVEF (%) | 0.97 | 0.96-0.98 | **<0.0001** |
| RVESV index (mL/m²) | 1.01 | 1.01-1.02 | **<0.0001** |
| RVEDV index (mL/m²) | 1.01 | 1.00-1.01 | **0.0110** |
| LA Volume index (mL/m²) | 1.02 | 1.02-1.03 | **<0.0001** |
| Presence of any LGE Pattern, n (%) | 1.44 | 1.06-1.96 | **0.0190** |
| Subendocardial Pattern, n (%) | 1.67 | 1.25-2.21 | **0.0004** |
| Non-Ischemic Pattern, n (%) | 1.28 | 0.96-1.71 | 0.0890 |
| Midwall Striae, n (%) | 1.19 | 0.83-1.72 | 0.3500 |
| RV Insertion Site, n (%) | 1.55 | 1.12-2.15 | **0.0084** |
| Midwall Patchy, n (%) | 0.869 | 0.50-1.53 | 0.6200 |
| Subepicardial, n (%) | 0.627 | 0.31-1.27 | 0.2000 |
| Diffuse, n (%) | 0.419 | 0.06-2.99 | 0.3900 |
| **Medications** | | | |
| ACE Inhibitor or ARB, n (%) | 2.89 | 1.57-5.31 | **0.0006** |
| Anti-Arrhythmic, n (%) | 1.79 | 1.04-3.09 | **0.0360** |
| Anti-Coagulant, n (%) | 2.32 | 1.74-3.08 | **<0.0001** |
| Anti-Platelet (non-ASA), n (%) | 1.16 | 0.80-1.69 | 0.4300 |
| ASA, n (%) | 1.27 | 0.96-1.69 | 0.1000 |
| Beta-Blocker, n (%) | 2.55 | 1.45-4.49 | **0.0011** |
| Calcium Channel Blocker (Dihydropyradine), n (%) | 1.33 | 0.89-1.98 | 0.1700 |
| Calcium Channel Blocker (non-Dihydropyridines), n (%) | 0.656 | 0.24-1.77 | 0.4000 |
| Digoxin, n (%) | 1.66 | 1.06-2.62 | **0.0280** |
| Loop Diuretic, n (%) | 4.28 | 3.19-5.73 | **<0.0001** |
| Thiazide Diuretic, n (%) | 1.09 | 0.65-1.82 | 0.7400 |
| K-Sparing Diuretic, n (%) | 2.07 | 1.55-2.76 | **<0.0001** |
| Entresto, n (%) | 1.28 | 0.82-2.02 | 0.2800 |
| Glucose Lowering, n (%) | 2.05 | 1.49-2.82 | **<0.0001** |
| Glucose Lowering (DPP-4 Inhibitors), n (%) | 2.6 | 1.33-5.07 | **0.0052** |
| Glucose Lowering (SGLT 2 Inhibitors), n (%) | 1.2 | 0.50-2.93 | 0.6800 |
| Insulin, n (%) | 2.11 | 1.37-3.27 | **0.0008** |
| Nitrates, n (%) | 2.66 | 1.99-3.55 | **<0.0001** |
| Statins, n (%) | 1.63 | 1.21-2.21 | **0.0014** |
| Smoking Cessation Agents, n (%) | 1.04 | 0.73-1.49 | 0.8300 |
| **Abbreviations:** ACE, angiotensin-converting enzyme; ARB, angiotensin II receptor blocker; BSA, body surface area; CAD, coronary artery disease; COPD, chronic obstructive pulmonary disease; EDV, end-diastolic volume; EF, ejection fraction; ESV, end-systolic volume; ICM, ischemic cardiomyopathy; LA, left atrial; LGE, late gadolinium enhancement; LV, left ventricular; NYHA, New York Heart Association; RV, right ventricular. | | | |

**Supplemental Figure 1:** Risk Score Nomogram for the prediction of HF hospitalization at 90-days, one-year, and two years.
